# Editorial: Psychological impact of COVID-19 on individuals: through active choices and passive adaption

**DOI:** 10.3389/fpsyg.2024.1356562

**Published:** 2024-02-06

**Authors:** Wanshan Wu, Haijing Liu, Shanshan Liu, Jianchun Fang, Macro Chi Keung Lau, Cheng Yan

**Affiliations:** ^1^School of Economics, Zhejiang University of Technology, Hangzhou, China; ^2^Yangtze River Delta Innovation Center, Zhejiang University, Hangzhou, China; ^3^Department of Economics and Finance, Hang Seng University, Hong Kong, Hong Kong SAR, China

**Keywords:** editorial, impact, COVID-19, choices, adaption

The emergence of the COVID-19 outbreak led to a global socio-economic crisis and widespread psychological distress (Pedrosa et al., 2020; Serafini et al., 2020). Various studies have examined the effects of the pandemic on psychological wellbeing, targeting diverse populations (e.g., Jiang, 2020; Di Riso et al., 2021). However, limited research has focused on the adverse effects on vulnerable groups. In addition, amidst the stress caused by the global health crisis, collective concern centers on how to cope with this pandemic effectively. Previous works have highlighted the role of family support and better education (e.g., Hu et al., 2022; Liu et al., 2023). More studies are needed to explore the potential mechanisms to mitigate the detrimental impact of the pandemic, including both active choices and passive adaptation.

This Research Topic aims to present a comprehensive overview of opportunities and gaps in the field, offering insights and perspectives for the future. That is, it focuses on exploring how the uncertain environment caused by the COVID-19 pandemic affects individuals' work, lifestyles, and mental wellbeing. It delves into how people respond actively and adapt passively to the challenges posed by the pandemic. We offer a Research Topic of sixteen articles encompassing both review and research studies. These manuscripts provide a better understanding of the diverse effects of COVID-19 on individuals across various nations, income brackets, and sectors, revealing heterogeneity in the impact.

Firstly, some studies have examined the detrimental effect of the COVID-19 pandemic lockdown on the psychology of children and adolescents. Specifically, Jin et al. have found that students in secondary and high school from northwest China experience prominent anxiety sensitivity. Factors such as gender, education level, family involvement in anti-COVID-19 efforts, illness during the lockdown, and specific fears related to the pandemic contribute to elevated anxiety sensitivity levels. Limone and Toto have documented a positive association between excessive digital technology usage and negative psychological and emotional outcomes for individuals aged 14–18. Therefore, adolescents within these age groups face adverse psychological consequences when their digital involvement rises during the global pandemic. Moreover, Pena-Shaff et al. have revealed that caregivers' anxiety increases following the outbreak of COVID-19 and thus has a negative effect on their children's emotional states. In addition, English et al. have highlighted the unique challenges of COVID-19 stress faced by Chinese international students, including fear of infection and acculturation-related stress.

Secondly, some studies have investigated the changes in women's psychological state during the COVID-19 pandemic. Specifically, Omiya et al. have found that almost half of Japanese women in this study experience a decrease in sense of coherence during the pandemic. However, good mental and physical health conditions, rich social capital, and having a job at the pre-pandemic stage can help mitigate the adverse effect. Phutong and Thaithae have revealed moderate anxiety levels in pregnant women during the pandemic outbreak in Thailand, with health literacy and social support identified as significant predictors.

Thirdly, some studies have focused on the adverse impact on elderly people and patients. Mameli et al. have shown that the COVID-19 pandemic has adverse effects on the mental health of individuals with Parkinson's disease, attributed to disrupted healthcare services, loss of routine activities and support, and reduced physical activity.

Fourthly, COVID-19 pandemic can result in more serious problems such as suicide. Bahk et al. have found that the increased levels of social distancing caused by the pandemic are associated with worsening depressive and anxiety symptoms, higher suicide risk, and heightened psychological distress among participants in South Korea. Besides, based on the sample from the northeastern Mexican border population, Villarreal Sotelo et al. have further supported this finding. Moreover, Ausserhofer et al. have suggested that emotional burden (i.e., depression, anxiety, and stress) can increase the probability of infection and have shown an interplay involving psycho-neuroendocrine and immune mechanisms. Put differently, the mental disorder resulting from the global health crisis can lead to higher infection by SARS-CoV-2, which is the virus responsible for causing this disease.

While available evidence indicates that mental health can be adversely affected across all age groups during the global pandemic, some studies have explored factors that can alleviate the detrimental effects on people's psychological wellbeing. Firstly, the negative impact on children's emotions can be alleviated through factors such as health literacy, wise reasoning, family support, and social support. Specifically, Huang L. et al. have suggested that factors like a supportive family environment (e.g., moderate financial and emotional support) and higher parental education contribute to better mental health. For Chinese international students, traditional coping strategies such as acceptance, reframing, and striving—methods involving accepting trauma, adjusting to reality, and reinterpreting tragic events—along with family support, do not alleviate the anxiety induced by COVID-19 stress. However, the application of wise reasoning, which signifies adept decision-making in intricate circumstances, has proven to be effective in mitigating this anxiety.

Furthermore, some studies have further explored how to decrease the negative impact on other age groups. In particular, Huang Y. et al. have found that the presence of meaning and self-esteem can substitute the role of social support in reducing death anxiety. Yang et al. have documented that higher income has a positive effect on mental health during the pandemic by investigating China's families. Additionally, factors such as the number of cigarettes smoked per day, education level, marital status, and exercise frequency also contribute to mental health. Moreover, Zheng et al. have identified a positive association between the perceived risk of unemployment and depression, with a greater impact on rural adults compared to urban adults.

Finally, except for mental health, Ma et al. have investigated the social-economic effect following the outbreak of the global pandemic. Their analysis has identified new factors contributing to financial vulnerability, including the pandemic itself, the digitization of the economy, financial literacy, addiction to digital technology, and changes in financial behavior. In addition, Phulkerd et al. have found food insecurity prevalence in Thailand during the COVID-19 pandemic.

In conclusion, this Research Topic presents a comprehensive overview of the various aspects related to the adverse impact of the COVID-19 pandemic on individuals' mental health, social dynamics, financial vulnerability, and food security across diverse populations and regions. In terms of mental health, health literacy, wise reasoning, family and social support, education level, as well as well-paid jobs can help people actively and passively adapt to the pandemic situation. Besides, the presence of meaning and self-esteem can also play a pivotal role in mitigating the adverse impact. Moreover, special attention should be directed toward vulnerable social groups such as girls, younger students, women, and those from low-income families. However, how to mitigate the adverse effect on the society and economy remains unsolved. In this case, all the research highlights the need for targeted interventions and policy considerations to address the challenges posed by this global health crisis. Future research can further investigate individuals' work, lifestyles, and socio-economic challenges caused by the COVID-19 pandemic, exploring effective coping strategies for navigating the uncertainty in this context. Finally, additional long-term studies are needed to investigate its lasting effects.

## Author contributions

WW: Funding acquisition, Writing – original draft. HL: Formal analysis, Writing—original draft. SL: Writing—original draft. JF: Conceptualization, Data curation, Writing—original draft. ML: Writing—original draft, Conceptualization, Data curation, Investigation, Methodology, Software. CY: Conceptualization, Formal analysis, Project administration, Resources, Supervision, Validation, Visualization, Writing—original draft, Writing—review & editing.
